# 

**Published:** 1841-10

**Authors:** 


### THE WESTERN JOURNAL OF MEDICINE AND SURGERY:

EDITED BY

DANIEL DRAKE, M. D.

AND

LUNSFORD P. YANDELL, M. D.

PROFESSORS IN THE LOUISVILLE MEDICAL INSTITUTE.

NO. XXII.—OCTOBER, 1841.

VOL. IV.—NO. IV.

LOUISVILLE, KY.

PUBLISHED MONTHLY, BY PRENTICE & WEISSI[illegible]

PROPRIETORS AND PRINTERS.

1841.

*This Number contains 4 sheets—Postage under* 100 *miles* 6 *cents, over* 100 [illegible]

